# 3D-printed setup for ocular drug delivery evaluation on ex vivo porcine whole eye

**DOI:** 10.1038/s41598-025-12081-9

**Published:** 2025-07-31

**Authors:** Felipe M. González-Fernández, Daniele A. Cauzzi, Annalisa Bianchera, Paolo Gasco, Cristina Padula, Patrizia Santi, Sara Nicoli, Silvia Pescina

**Affiliations:** 1 ADDRes Lab, Department of Food and Drug, University of Parma, Parco Area delle Scienze, 27/a, Parma, 43124 Italy; 2 Nanovector S.r.l., Via Livorno, 60, Turin, 10144 Italy; 3 Department of Chemistry, Life Science and Environmental Sustainability, University of Parma, Parco Area delle Scienze, 17/A, Parma, 43124 Italy

**Keywords:** Drug delivery, Pharmaceutics

## Abstract

Reaching the back of the eye without invasive intraocular injections is still nowadays challenging. Luckily, the nanotechnology can help in improving the effectiveness and the patient compliance and, ultimately, the therapeutic success rate. The development and characterization of ophthalmic (nano)formulations benefit from the implementation of ex vivo models, that allow to disclose the formulation behaviour once in contact with biological tissues and consequently its optimization. Ex vivo ocular models help to minimize the number of promising formulations deserving to be investigated in in vivo animal models, in compliance with the 3Rs principle, and therefore their availability is of paramount importance. In this research work, we present a 3D-printed reusable device designed to be used in a whole porcine eye bulbs setup comparing it with the Franz-cell. The device, produced by a Masked Stereolithography Apparatus, consists of two components, the Ocudonor, acting as a donor chamber for the formulation, and the Ocutainer, which simultaneously contains up to three ocular bulbs. The porcine eye setup was validated using a dexamethasone-loaded nanostructured lipid carriers for periocular administration and intended for the treatment of the posterior segment of the eye. The porcine whole eye model proved capable of preserving eye structure integrity for at least 18 hours and, unlike the Franz-cell, enabled the evaluation of the drug’s lateral diffusion along the eye bulb.

## Introduction

Treatment of conditions affecting the back of the eye is a cumbersome task, due to the limited accessibility to the inner eye^1^. A plethora of nanoparticulate systems have been studied over the past decades because their unique characteristics can help overcome the static, dynamic, and metabolic barriers of the eye^2^.

Formulative studies, aiming to evaluate and optimize the ophthalmic nanoparticulate systems, benefit from the use of ex vivo animal models. In fact, the contact between nanoparticulate systems and ocular tissues (i.e., conjunctiva, cornea, sclera and choroid) allows to quickly screen the formulation candidates in biorelevant conditions. The most common, reproducible, and simple setup is the Franz-cell which, in turn, can only evaluate unidirectional permeation and retention within isolated tissue samples^3–5^. To collect information about distribution within ocular tissues some examples on the use of ex vivo whole eyes with^6^ or without^7–9^ perfusion have been already reported. However, the use of isolated whole eyes is challenging, being associated with the difficulty of maintaining the integrity of ocular barriers for a time compatible with permeation and retention experiments.

Developing a simple, non-perfused whole eye ex vivo model is of primary importance for characterization and screening of formulation candidates during preclinical stages. The required high level of precise customization to develop this model can be achieved using an affordable and convenient tool such as the 3D printing. The 3D printing is now regarded as an “umbrella term” encompassing a variety of additive manufacturing techniques. The common factor among them is the process of designing a digital three-dimensional object using computer-aided design (CAD) software, which is then “sliced” into multiple layers. These layers are then printed sequentially to build a physical three-dimensional object.

The application of 3D printing in preclinical research has been steadily increasing over the past years^10–13^. Among others, a simple lab setup to analyse protein adsorption and resorption from ocular contact lenses was developed with stereolithography (SLA) technology^14^; moreover, two fused deposition modelling (FDM) printed devices, have been proposed to modify cuvettes conventionally used for the measurement of oxygen permeability of contact lenses^15^. FDM has also been used to evaluate drug release from soft contact lenses by manufacturing an ocular model that simulates the physiological blinking^16^. The model was further improved and printed with SLA technology to ensure watertightness^17^. A novel in vitro dissolution model, the Eye Flow Cell, for drug release characterisation of intravitreal implants, has also been reported. In combination with a simulated vitreous, composed of agarose (shell) and polyacrylamide (core), the release setup allowed for a better mimicking of physiological conditions with more accurate results when compared to standard USP (United States Pharmacopeia) apparatuses used for drug release studies^18^.

We hereby present the development of a novel ex vivo setup based on whole porcine ocular bulbs. In addition, nanostructured lipid carriers loaded with dexamethasone were developed to demonstrate the validity of the setup.

## Results

### The 3D-printed Ocudonor and Ocutainer devices

**Fig. 1:**
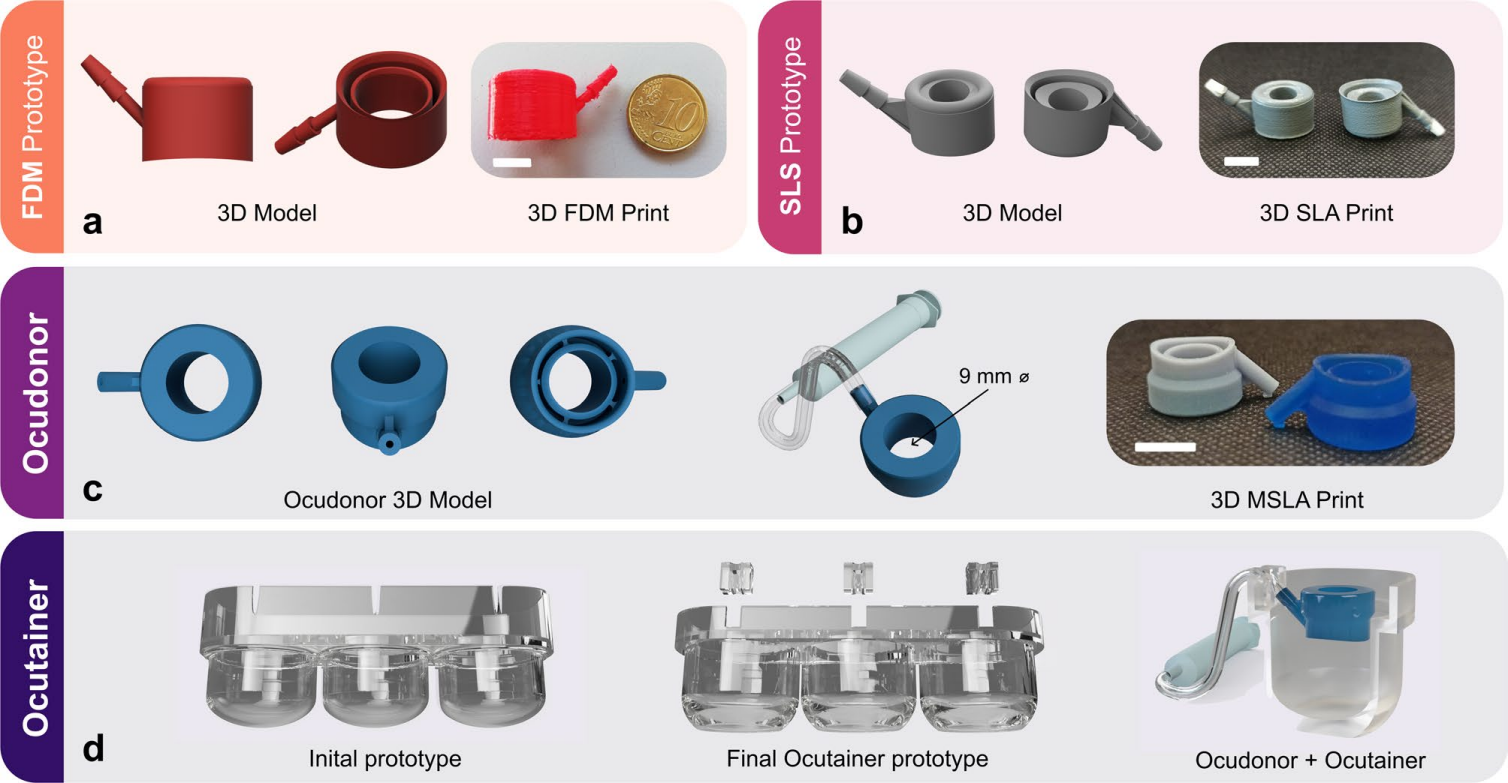
Development process of the 3D-printed Ocudonor and Ocutainer devices from the initial prototypes printed using fused deposition modelling (FDM) technique (**a**) and Selective Laser Sintering (SLS) techniques (**b**) up to the final devices produced via the masked stereolithography (MSLA) technique (**c**, **d**). White scale bars correspond to 1 cm.

Three different 3D-printing techniques were evaluated, and the complexity of the designs was adjusted to the printing resolutions of each technique. The initial FDM prototype presented an oval permeation area, but the printing resolution remained low due to the large diameter of the extruded PLA filament. Without any postprocessing, the resulting device was not airtight and would not adhere to the ocular bulb (Fig. 1a).

The SLS prototype (Fig. 1b) maintained the initial oval shape but presented a circular permeation area of 9 mm diameter (0.6 cm^2^). The device was printed in polyamide 12 using a SLS system (externalized production). Despite the high printing resolution, the 3D-printed device remained highly porous, and the polyamide material might entrap the drug to a non-negligible extent, as also demonstrated by its use as textile fibre that greatly absorbs dyes^19^. Acrylic spray coating scarcely improved the air tightness. Furthermore, application onto a whole porcine eye also failed due to the high thickness of the internal ring wall between the vacuum chamber and the permeation area.

The oval shape of the FDM and SLS prototypes was modified to a circular shape with a circular permeation area, similarly to a classical donor chamber in Franz-type diffusion cells (Ocudonor, Fig. 1c). The rims of the inner ring were mildly bevelled to maximize the grip while minimizing the possibility of damaging the tissue. The outer ring was rounded and twisted following the natural curvature of the ocular bulb at the equatorial point. Thanks to its lateral arm, acting as a vacuum tube, Ocudonor can be easily connected to a 5 mL syringe, which serves as a suction system to attach the device to the ocular surface. The Ocudonor device was designed and printed using a small scale MSLA (masked stereolithography apparatus) liquid resin printer which reaches a 47 μm x 47 μm xy resolution and a 50 μm layer thickness.

The Ocutainer device was developed to maintain adequate temperature and humidity values throughout the whole experiment (Fig. 1d). The Ocutainer accommodates up to three ocular bulbs which remain isolated from each other in single cups. The initial prototype only considered a simple crevice which would hold the vacuum tubing coming from the Ocudonor. The final Ocutainer prototype presents an insert that acts as an inlet for the vacuum tubing maintaining the 45° angle and allowing for free movement and action of the syringe without any risk of detachment from the scleral surface.

### The whole porcine eye ex vivo setup using the Ocudonor and Ocutainer devices

**Fig. 2:**
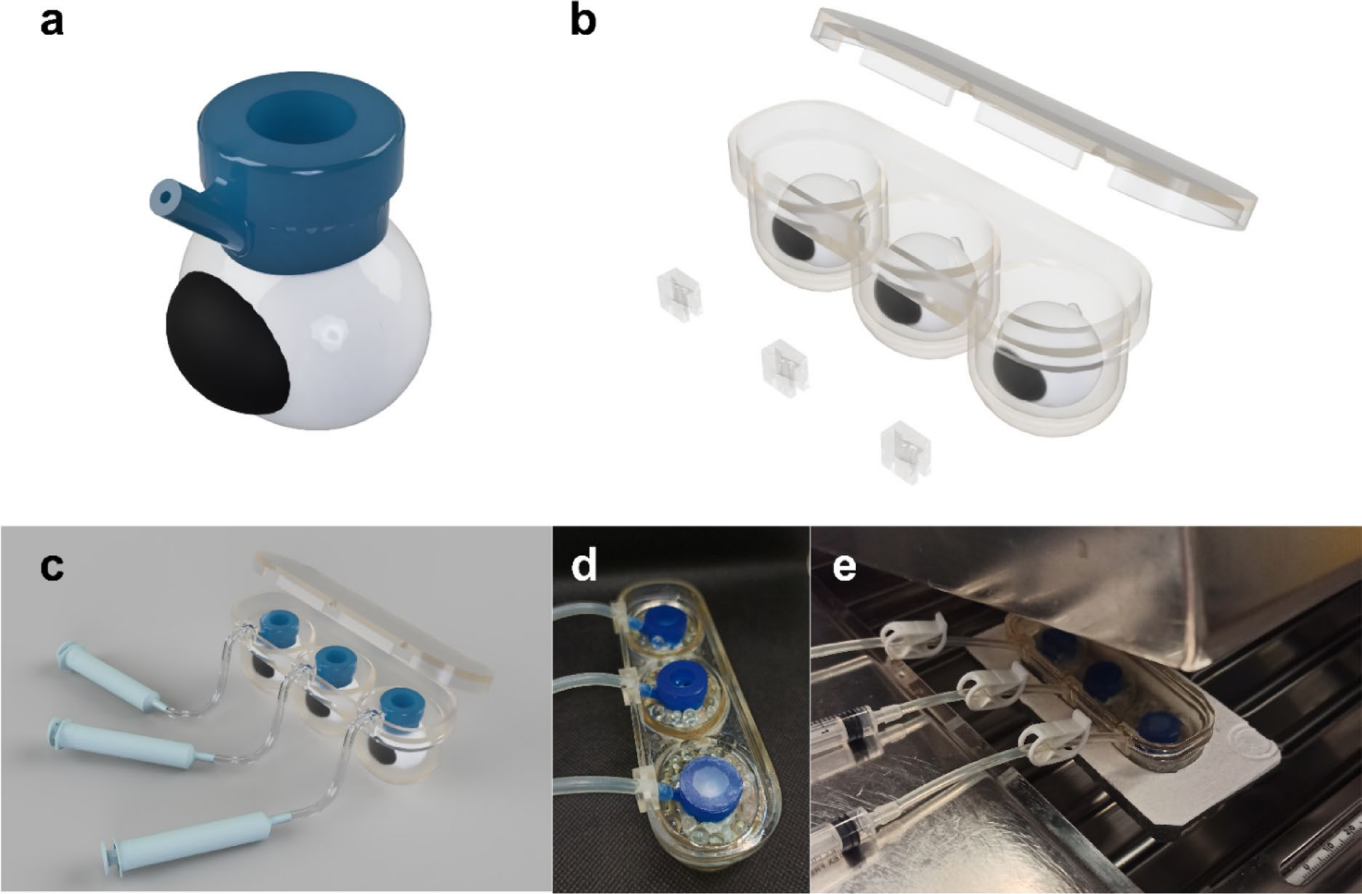
Images of the whole eye ex vivo setup: (**a**) 3D rendering of the Ocudonor device and expected placing on the scleral surface; (**b**) 3D rendering of the Ocutainer device, including the lid and the inserts for tubing; (**c**) 3D rendering of the whole setup, including the syringes for vacuum adhesion; (**d**) close-up image indicating the glass beads and parafilm coverage of each Ocudonor and (**e**) setup immersed in the water bath with a closing lid. 3D renderings generated with Autodesk Fusion 360 software.

The 3D-printed ex vivo whole swine eye setup is depicted in Fig. 2. A lid allows the preservation of a constant atmosphere which is further maintained by the lid of the water bath. The whole setup can be placed inside an expanded polystyrene support for floating.

The setup ability to maintain tissue hydration for up to 18 h was tested, and a weight loss ranging from 0.13 to 0.99% (*n* = 6) was observed.

### Dexamethasone-loaded nanostructured lipid carriers (Dex-NLC)

Physicochemical characterization of the freshly obtained dexamethasone-loaded NLC showed at day one a Z-average diameter of 98.2 ± 3.8 nm and a PDI of 0.258 ± 0.023. After one month, the Z-average diameter of the nanoparticles was 172.9 ± 39.0 nm and the PDI 0.228 ± 0.026, which indicates a possible nanoparticle aggregation. This is mainly explained by the nearly neutral surface charge of the NLC, as indicated by the zeta-potential values of -2.93 ± 0.25 mV which did not strongly change over one month of study (-3.72 ± 0.19 mV).

**Fig. 3:**
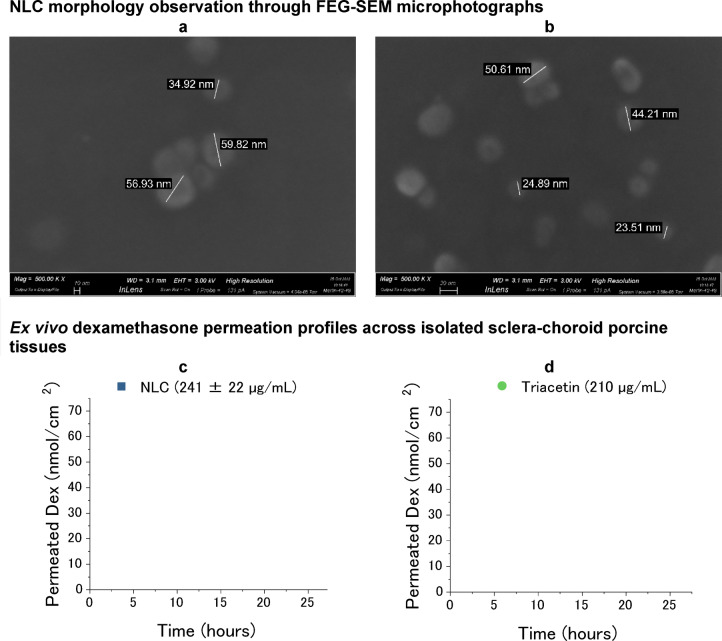
Characterization of the developed NLC. (**a**, **b**) FEG-SEM images obtained at different magnifications of air-dried Dex-NLC diluted 1:100; (**c**) permeation profiles across sclera-choroid (SCh) of Dex from Dex-NLC, donor concentration 241 ± 22 µg/mL (corresponding to 614 ± 57 µM) and (**d**) Dex diffusion across sclera-choroid when a 210 µg/mL solution in triacetin (corresponding to 535 µM) is used as donor.

Morphology of the NLC was determined by FEG-SEM microscopy on air-dried 1:100 diluted samples and photomicrographs at different magnifications were obtained (Fig. 3a,b) where the nanoparticles appear spheroidal.

The dexamethasone diffusion across the double barrier sclera-choroid over 24 h were obtained using Franz cells: permeation profiles of dexamethasone from Dex-NLC and a triacetin solution, chosen as reference, are reported in Fig. 3c,d, respectively.

When Dex was applied as a solution in triacetin the calculated apparent permeability coefficient P_app_ was only 1.5 ± 0.9 * 10^− 7^ cm/s, one order of magnitude lower than the obtained value when dexamethasone was delivered through the NLC (1.1 ± 0.2 * 10^− 6^ cm/s). This result finds an explanation in the different affinity of the drug for the NLC with respect to the oil. The solubility of plain dexamethasone in triacetin is approximately 1 mg/mL, but enough to justify a limited trans-scleral diffusion in comparison to NLC encapsulated drug. The affinity for triacetin was confirmed also by retention data, shown in Table 1.

**Table 1:** Retention within fresh porcine tissues after 24 h contact at 37 °C in Franz-cells with Dex-NLC and Dex dissolved in triacetin (S = sclera; ch = choroid; SCh = sclera + choroid).

		Dex-NLC (241 µg/mL = 614 µM)^a^ (*n* = 8)	Triacetin (210 µg/mL = 535 µM) (*n* = 4)
Sample	Tissue	Dex (nmol/g tissue)	Dex (nmol/g tissue)
SCh	S	132.2 ± 27.7	11.6 ± 1.8
	Ch	101.1 ± 21.1	0.0 ± 0.0

^a^Average donor concentration.

Dexamethasone is significantly retained within both the sclera and the choroid, suggesting the formation of a reservoir and thus a presumable sustained drug release over time.

### Performance of the Dex-NLC in the ex vivo whole eye Ocudonor-Ocutainer setup

**Fig. 4:**
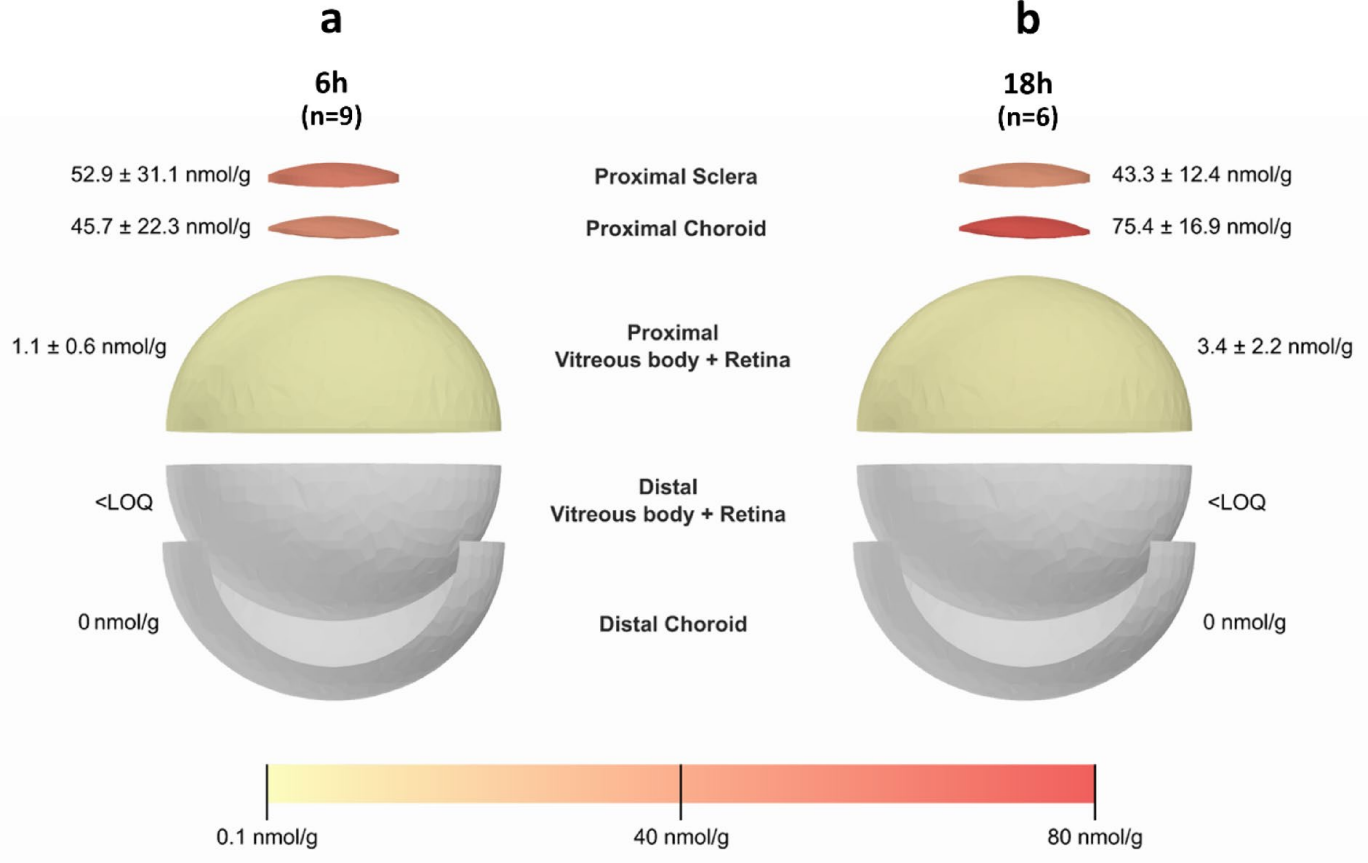
Dexamethasone retained within different ocular tissues (reported as mass of drug per mass of tissue) in *ex vivo* whole eye using the Ocudonor-Ocutainer setup. Data were collected after a contact of 6 h (*n* = 9, panel **a**) and 18 h (*n* = 6, panel **b**) of Dex-NLC (580.83 ± 31.86 µM).

Dex-NLC were further tested ex vivo on whole porcine eyes using the Ocudonor-Ocutainer setup. Dexamethasone was extracted and quantified from ocular tissues after 6 and 18 h of contact, and the obtained data are presented in Fig. 4.

After six hours, a gradient is clearly observable for dexamethasone concentration following the order: proximal sclera > proximal choroid > proximal vitreous-retina > distal vitreous-retina > distal choroid. When increasing the contact time to 18 h, the sclera in direct contact with the formulation appears to reduce its drug content and a shift between the sclera and proximal choroid is observed.

The reported values refer only to a small portion of the sclera and proximal choroid corresponding to the permeation area, that is the 0.6 cm^2^ circular area delimited by the Ocudonor device, which is the same permeation area of the Franz-type cells previously used. The dexamethasone concentration detected within the sclera after 18 h in the whole eye ex vivo model (Fig. 4) is considerably lower than the concentration reached in 24 h using the Franz-cell setup (Table 1), being 43.3 µg/g vs. 132 µg/g, respectively. This difference can be mainly attributed to a lateral diffusion of dexamethasone occurring, when using Ocudonor, in both sclera and choroid. In a Franz-cell, lateral diffusion is not feasible and the drug flux, driven by the concentration gradient, necessarily follows a single direction. On the contrary, when the whole ocular bulb is used, diffusion can occur also laterally, explaining the progressive decrease in concentration at the permeation area.

## Discussion

Ex vivo ocular studies are an essential step in the development of ophthalmic formulations. Data collected in permeation and/or retention experiments provide more predictable results of in vivo behaviour, fundamental information for disclosing formulation behaviour in biorelevant conditions.

Porcine eyes are anatomically and physiologically similar to human eyes and are therefore a valuable ex vivo model. Considering that they are usually discarded as by-products of the meat production industry, their implementation in preclinical research also complies with the 3Rs principle (Replacement, Reduction and Refinement) in animal experimentation.

However, since experiments are generally performed with isolated tissue sections in a Franz-cell setup, information about multidirectional ocular drug distribution is missing and a more complex whole eye model is needed. The development of an advanced ex vivo ocular model based on whole ocular bulb faces two major limitations. Firstly, the spheroidal shape of the eye hinders the application of liquid or semisolid formulations on a defined site for prolonged times without unspecific spreading over the ocular surface. Secondly, ocular bulb orientation and hydration levels should remain unvaried throughout the experiment, and therefore an adequate temperature and humidity range must be ensured. The ex vivo ocular model presented hereby aims to overcome both these limitations. The strategy proposed consisted in the design and 3D-printing of two reusable devices tailored to the porcine eye anatomy: Ocudonor and Ocutainer.

The porcine eye was 3D-modelled from multiple bidimensional images of frozen eyes by photogrammetry (i.e., achieving precise measurements via photo-based method) and further adjusted using mean values of porcine eye measurements available in the literature^20,21^. This served as a template for the construction of prototypes with increasing complexity, mainly limited by the printing technique evaluated: from low-resolution FDM to non-airtight SLS prototypes (Fig. 1a,b). The best compromise between high definition and low air leak was reached using an inexpensive MSLA resin printer (Elegoo Mars).

The Ocudonor applicator (Fig. 2) was designed to contain up to 0.5 mL of formulation at a defined circular permeation area of 0.6 cm^2^ thus enabling to establish a “starting point” onto the scleral surface from which the distribution phenomena will start. In addition, the device should exert a tight adherence onto the scleral surface at the equatorial level (i.e., concentric to the vertical axis of the eye) for up to 18 h with little to no damage to the tissue. Hessburg-Barron corneal trephines are surgical instrumentation commonly used in corneal transplantation procedures^22^. The device firmly attaches around the cornea thanks to an annular vacuum chamber in which a small negative pressure is applied with the help of a syringe. A similar vacuum annulus is commercially available as a medical device for corneal delivery of riboflavin by iontophoresis for the treatment of keratoconus in both humans (Iontofor-CXL^®^, Fidia Farmaceutici S.p.A., Italy)^23^ and animals (Iontoforvet^®^, OPIA Technologies S.A.S., France)^24^. This minimally invasive principle of a vacuum annulus was adopted also for the design of the Ocudonor but adjusted to the porcine scleral curvature. The drug applicator was termed Ocudonor since it acts as a donor chamber that contains the dosage form (liquid, semisolid or solid) under study.

The designed Ocutainer stabilizes the eye-Ocudonor-syringe assembly and, what is more, ensures adequate temperature and humidity values for up to 18 h.

The Ocudonor-Ocutainer setup was tested with a previously proposed NLC that had demonstrated transscleral delivery of the anti-inflammatory corticosteroid dexamethasone^25^. Dexamethasone concentration inside the proximal vitreous body is three times higher after 18 h, indicating continuative progress of the diffusion across tissues, and corresponds to 3.4 nmol/g. This value is consistent with the drug concentration required for producing a therapeutic effect in the back of the eye, namely 2.5 nmol/g^26^.

It is important to emphasize that the current ocular model lacks dynamic barriers, and therefore, the accumulation data obtained do not account for the clearance role of choroidal circulation. Nevertheless, the strength of this model lies in its ability to screen various formulation candidates with respect to their behaviour towards static and metabolic barriers. Subsequently, only selected formulations will proceed to in vivo evaluation, significantly contributing to the reduction in the number of animals used, in line with the 3Rs principle. The evaluation of the screened formulation on in vivo model will be necessary to clearly highlight the relevance of the model with respect to translational aspects. Moreover, even if the model is validated for 18 h, further studies will be conducted to extend the experiments to at least 24 h. Another key consideration is the possible drug absorption by the Ocudonor: to rule out any possible interference, the material chosen for printing should undergo preliminary testing.

Finally, although the system is intended for studying trans-scleral permeation, it can be easily redesigned and adapted to investigate ocular drug distribution following corneal application.

## Methods

### Materials

Dexamethasone (Dex; molecular weight 392.5 g/mol; water solubility 89 µg/ml; LogP 1.9^27^) and tyloxapol were purchased from Sigma Aldrich (Taufkirchen, Germany). Triacetin (glycerol triacetate) was provided by TCI Europe N.V. (Zwijndrecht, Belgium). Imwitor^®^ 491 (I491, glycerol monostearate (GMS); ≥90% content in monostearate) was a gift from IOI Oleo GmbH (Hamburg, Germany). Vitamin E-derived tocopherol polyethylene glycolsuccinate (TPGS; MW *approx.* 1513 g/mol) was kindly donated by PMC ISOCHEM (Vert-Le-Petit, France). Saline solution (9 g/L NaCl) and phosphate-buffered saline (PBS; 0.19 g/L KH_2_PO_4_, 2.37 g/L Na_2_HPO_4_, 8.8 g/L NaCl; pH 7.4 by adding 85% H_3_PO_4_) were prepared using ultra-pure water (Arium^®^ Comfort Sartorius, Goettingen, Germany).

All other chemicals were of analytical grade.

### 3D printing

3D modelling was performed with the CAD software Autodesk^®^ Tinkercad (Autodesk^®^ Inc, California, USA); models meshes were adjusted and repaired with the open source packages MeshLab and Autodesk Meshmixer; pre-processing and slicing for 3D printing with Chitubox^®^ (Guangdong, China) software. FDM and SLS prototypes manufacturing was externalized. MSLA 3D printing occurred on an Elegoo Mars UV Photocuring LCD MSLA printer (Elegoo, Shenzen, China) with eSun Hard-Tough blue resin (Shenzhen eSun Industrial Co., Ltd, Shenzen, China) and Elegoo ABS-like clear Photopolymeric rapid resin (405 nm). Printing settings were adjusted according to manufacturer specifications. The printed devices were left to drip the excess resin overnight, then cleaned using a paper cloth, then washed by full immersion in reagent grade isopropanol while sonicating, two times changing the isopropanol. Finally, they were thoroughly washed with distilled water and dried using a stream of compressed air. The curing was carried out using 4 × 9 watts UV fluorescent lamps. The curing time was 5 min. Two devices have been developed: the eye applicator allowing for contact between the eye and the dosage form, named Ocudonor, and the eye container, called Ocutainer. After photopolymerization, the Ocutainer was coated with transparent epoxy resin (SigWong^®^, China) to ensure water tightness.

### NLC preparation and characterization

NLC were produced by modifying a previously reported method^25^. Briefly, 125 mg tyloxapol, 75 mg I491, and 50 mg triacetin were warmed to 65 °C and, after 5 min, 4.5 mL ultra-pure deionized water were added under magnetic stirring. The warm microemulsion was then added dropwise into 4.5 mL ultra-pure deionized water at 4 °C under sonication (Branson 2510 Ultrasonic Bath Sonicator, Branson Ultrasonics, Danbury, CT, USA), up to 10 min. Drug-loaded NLC were prepared by adding 2 mg dexamethasone to the initial warm microemulsion: the final dexamethasone concentration was 235 ± 20 µg/mL (corresponding to 600 ± 50 µM).

#### Determination of size, polydispersity index and zeta potential

Nanoparticle size and polydispersity index (PDI) were measured by triplicates on a Malvern Zetasizer Nano ZS (Malvern Panalytical Ltd., Malvern, UK): each sample was previously diluted 1:50 with ultra-pure water.

Zeta potential was obtained by dilution (1:50) in a 10 mM NaCl solution in water, also by triplicates.

#### Morphology analysis: FEG-SEM

Nanoparticles were observed on a Field Emission Gun Scanning Electron Microscope working at an acceleration voltage of 3.0 kV (FEG-SEM; ZEISS Merlin^®^ FEG-SEM microscope, Oberkochen, Germany). Samples were diluted 1:100 with ultra-pure deionized water and 5 µL were directly deposited onto SEM specimen stubs and dried in a desiccator for 24 h before analysis. No sputter coating was required. Photomicrographs were obtained at 500k magnification.

#### Ex vivo permeation and retention using Franz-type diffusion cells

Freshly excised swine eyes (Landrace and Large White breeds, both female and male; 10–11 months of age; 145–190 kg) were obtained from a local abattoir (Macello Annoni S.p.A., Busseto, Italy) and transported in saline solution at 4 °C. Ocular adnexa were removed, and the eye was incised with a scalp in the perilimbal region. The anterior segment and vitreous body were removed, as well as the retinal pigmented epithelium (RPE), to obtain the sclera-choroid eyecup. The eyecup was divided into two halves following the ciliary arteries and the optic-nerve-free half (SCh) was mounted on a 9 mm diameter vertical diffusion cell. The donor chamber was filled with 0.2 mL of the studied NLC within 48 h from the preparation (Dex concentration 241 µg/mL), and the receptor chamber contained *approx.* 4 mL of a 0.5 mM Vitamin-E-TPGS solution in PBS. The solubility of dexamethasone in the 0.5 mM Vitamin-E-TPGS solution in PBS was determined beforehand (103.5 ± 1.8 µg/mL; 25 °C). As a reference, a Dex 210 µg/mL solution in triacetin was used.

Dexamethasone permeation curves across the sclera-choroid (SCh) barrier were built by sampling 0.3 mL of receiving solution at different time points until 24 h, replacing the same volume with fresh media. The amount of Dex diffused across sclera-choroid (nmol/cm^2^) was plotted against time (hour): the slope of the regression line at steady state is the trans-scleral flux J_SS_ (nmol/cm^2^ h). Then, the apparent permeability coefficient P_app_ (cm/s) was calculated from the following equation:

1 $$P_{{app}} = J_{{ss}} /C_{d}$$

where C_d_ (µM) is the concentration of the donor solution.

The accumulation of the drug in the sclera and choroid was investigated separately following an extraction method previously validated^25 ^with ≥ 90% drug recovery. Briefly, the 9 mm diameter permeation areas were precisely punched, and the thin choroid was separated from the sclera with tweezers. All the samples were weighed. The sclera was placed into 1 mL of an extracting mixture containing acetonitrile and water at a 65:35 ratio (v/v) and left for two hours with eventual vortexing. Choroid was extracted in the same conditions but using only 0.5 mL of extraction mixture. A solution of dexamethasone in triacetin (0.3 mg/mL) was used as a reference.

### Validation of an extraction method from vitreous body and retina

Approximately 2 g of frozen vitreous body, including retina (VR), equivalent to half the volume of the vitreous body in a porcine eye, were transferred into 8 mL polypropylene tubes. 20 µL of 2 mg/mL dexamethasone dissolved in ethanol 90% (v/v) were added. After 30 min, the sample was liquefied with a sonicating probe at a constant frequency of 30 kHz (Amplitude 100% (180 μm), Cycle 1; UP100H ultrasonic Processor, Hielscher ultrasonics, Teltow, Germany) for 1 min. Aliquots of 0.35 mL from the digest were transferred into 1.5 mL Eppendorf^®^ tubes and 0.65 mL of acetonitrile were added. VR was left in contact with the extracting mixture for 2 h at room temperature with eventual sample vortexing. Samples were centrifuged at 12,000 rpm for 5 min and the supernatant analysed with HPLC for dexamethasone quantification. To check the presence of interferences from the tissue, VR without the drug (blank samples) was subject to the same extraction procedure. The amount of dexamethasone extracted from VR using this method is 99.9 ± 4.6%.

### Retention experiments on whole ocular bulbs

Three fresh (less than 4 h after slaughter) ocular porcine bulbs were placed into the Ocutainer (the eye container) and the Ocudonor (the device acting as the donor for dosage form) was firmly adjusted onto the distal half of the sclera (with respect to the optic nerve) and between both superior vorticose veins, keeping it in place by a vacuum, set with the use of a syringe (vacuum system). 200 µL of the formulation were added to the donor compartment and sealed. 2 mm diameter glass beads were placed between the ocular bulb and the Ocutainer walls to maintain the bulb in upright position. Approximately 3 mL of phosphate-buffered saline (PBS) were added to maintain tissue hydration throughout the experiment. The ex vivo setup was placed in a closed water bath (Memmert W350, Memmert GmbH, Schwabach, Germany) set at 37 °C, with a relative humidity of *approx.* 100%, for 6–18 h respectively.

After the experiment, formulations and donor chambers were removed, the donor area was accurately dried, marked with a paper filter disk and frozen (-20 °C) for at least 24 h. The frozen ocular bulbs were halved following the equatorial plane demarked by the ciliary arteries (corresponding to proximal and distal halves with respect to the donor area). For each half, the frozen vitreous body with attached retina was accurately weighted and submitted to ultrasonic homogenization and extraction, as previously described. On the proximal half, the permeation areas (9 mm) were punched, and both sclera and choroid were analysed, while, on the distal half, the whole choroid was retrieved (possible RPE was cleaned with filter paper). Extraction proceeded as previously described.

### Analysis

A previously validated HPLC/UV-Vis method was used for the quantification of dexamethasone^25^. Analysis was conducted with an Agilent 1260 Infinity apparatus (Agilent Technologies, Santa Clara, CA, USA). Separation occurred in a reverse phase column Nova-Pak^®^ C18 (4 μm, 3.9 x 150 mm; Waters, Milford, MA, USA) heated at 40 °C. The mobile phase, a mixture of acetonitrile and water in the ratio was 35:65 (v/v), was pumped at 1 mL/min. The detector was set at 246 nm and the retention proved to be 2.1 min.

Two calibration curves were used. For the extraction samples, standards were prepared in organic extracting mixture (acetonitrile/water, 65:35, v/v) and linearity was in the range of 0.2–10 µg/mL. In the case of permeation samples, solvent was 0.5 mM TPGS in PBS pH 7.4 and the range of linearity between 0.3 and 10 µg/mL. In all cases, the limit of detection (LOD) was ≥ 0.1 µg/mL and precision (expressed as relative standard deviation %) was lower than 2.5% for all studied concentrations while the relative error, indicative of method accuracy, remained in any case under 10%.

## Data Availability

Data are available from the corresponding author on reasonable request. Data here presented are part of F.M.G.F.’s PhD thesis “Development of ex vivo models for smart screening of novel colloidal ocular formulation”.
